# Factors associated with hookworm and *Schistosoma mansoni* infections among school-aged children in Mayuge district, Uganda

**DOI:** 10.1186/s12889-024-19092-7

**Published:** 2024-06-18

**Authors:** Eun Seok Kim, Moses Adriko, Kabarangira Christine Oseku, David Lokure, Emily L. Webb, Kalpana Sabapathy

**Affiliations:** 1 Department of Infectious Disease Epidemiology, London School of Hygiene and Tropical Medicine, London, UK; 2 Vector-Borne and NTDs Control Division, Ministry of Health, Kampala, Uganda; 3 World Vision Korea, Seoul, Korea; 4 Faculty of Health Science, Busitema University, Mbale, Uganda; 5 Information and Technology Sector, Kotido District Local Government, Kotido, Uganda

**Keywords:** Hookworm, Schistosomiasis, Risk factors, Mayuge, Uganda

## Abstract

**Background:**

Hookworm infection and schistosomiasis are two of sub-Saharan Africa's most common neglected tropical diseases. An annual mass drug administration (MDA) program against schistosomiasis and soil-transmitted helminths (STHs), including hookworm, has been implemented in Mayuge district, Uganda, since 2003 to date. However, hookworm and schistosomiasis remain prevalent in Mayuge district. Understanding the factors that predispose children to these infections in the context of MDA could inform interventions to reduce prevalence in Uganda and similar settings.

**Method:**

This cross-sectional study took place in 33 randomly selected primary schools in the Mayuge district from January to February 2022. Children in primary classes 4 or 5, in the selected schools provided single stool samples and completed questionnaires. Stool specimens were examined using the Kato-Katz method to determine the prevalence of hookworm and schistosomiasis. We performed univariable and multivariable logistic regression to assess the associations of each infection with potential risk factors.

**Result:**

A total of 1,617 students (mean age 12.1 years, 50.1% male) were enrolled. The prevalence of hookworm infection and schistosomiasis was 21.8% (95% confidence interval (CI): 19.8–23.9%) and 18.7% (95% CI: 16.8–20.7%), respectively. In multivariable analysis, longer water fetching time (over 30 min versus less than 30 min) and working daily in the soil were associated with increased odds of hookworm infection (adjusted odds ratio (AOR): 1.49, 95% CI: 1.13–1.96 and 1.37, 95% CI: 1.03–1.82, respectively). Higher odds of schistosomiasis were linked to proximity to water bodies within a one-hour walking distance (AOR: 1.84, 95% CI: 1.35–2.50), and not always washing hands before eating (AOR: 2.00, 95% CI: 1.50–2.67). Swimming, bathing, or washing in water bodies twice a week, compared to never, also increased schistosomiasis odds (AOR: 2.91, 95% CI: 1.66–5.13).

**Conclusion:**

Consistent with the mechanisms of acquisition, hookworm infection increased with exposure to soil, and schistosomiasis increased with exposure to unclean water. Our findings highlight the importance of Water, Sanitation, and Hygiene programs and strategies aimed at reducing exposure within the framework of Neglected Tropical Disease elimination programs.

**Supplementary Information:**

The online version contains supplementary material available at 10.1186/s12889-024-19092-7.

## Background

Hookworm is the commonest soil-transmitted helminth (STH), affecting 500 million people globally [1] and contributing to an estimated 4 million Disability-Adjusted Life Years (DALYs) worldwide annually [2]. Schistosomiasis affects more than 250 million people worldwide, with approximately 800 million, mainly children, being at risk [3, 4]. Schistosomiasis is associated with 1.4 million lost DALYs annually and with 280,000 deaths per year globally [5].

Adult hookworms residing in the host's gastrointestinal tract instigate blood loss, thereby giving rise to iron-deficiency anaemia and hookworm disease. Consequently, the severity of blood loss is directly proportional to the worm burden [6]. Persistent and chronic blood loss resulting from hookworm infection can contribute to long-term health consequences, manifesting as growth retardation, and mental and cognitive impairment [7, 8]. Also, hookworm infection in pregnant women can lead to adverse birth outcomes, including reduced birth weight and increased infant mortality rates [7, 9, 10]. *Schistosoma mansoni* infection causes intestinal schistosomiasis, producing severe sequelae such as hepatosplenomegaly with periportal liver fibrosis and portal hypertension, which progress from abdominal pain and bloody diarrhoea [11, 12]. Urogenital schistosomiasis caused by *Schistosoma haematobium* leads to haematuria, dysuria, hydronephrosis, female genital schistosomiasis and calcification of the bladder [13].

Hookworm infection and schistosomiasis are the two most common neglected tropical diseases (NTDs) in sub-Saharan Africa [14]. While not causing immediate fatal outcomes, these NTDs give rise to persistent and chronic health burdens that together contribute to combined DALYs due to hookworm and schistosomiasis higher than that of HIV/AIDS or malaria [3].

In Uganda, the estimated nationwide prevalence of hookworm infection and schistosomiasis is 7.7% and 25.6% respectively [15, 16], but schistosomiasis, as a consequence of *Schistosoma mansoni* infection, in particular is highly focal, over a third of the population are affected in several Ugandan districts [16]. Mayuge district is one such area and as a consequence of the high prevalence in this district, an annual mass drug administration (MDA) program against STH and schistosomiasis targeting school-aged children has been implemented since 2003. In addition, World Vision, funded by Korea International Cooperation Agency (KOICA), implemented the Mayuge NTDs Elimination (MANE) project between 2019 and 2022, a comprehensive NTD elimination program targeting STHs and schistosomiasis in Mayuge district. The MANE project involved annual MDA, water, sanitation and hygiene (WASH), and awareness increasing programs for community members and health workers.

In the MANE baseline survey, a total of 1,123 school-aged children (SAC) were enrolled in July 2019, the prevalence of hookworm and *Schistosoma mansoni* infection was 15.1% and 27.2% respectively, whereas other STHs were less than 1% [17]. The cure rate (CR) for hookworm by single-dose albendazole was approximately 78% [18] and CR for schistosomiasis by standard praziquantel (PZQ) dose of 40 mg/kg was over 70% [19], and there has been high program coverage of MDA for STH (77%, 81%, and 75% in 2012, 2014, and 2016 respectively) and for schistosomiasis (85%, 65%, and 72% in 2012, 2014, and 2016), respectively. As such, the continued burden of hookworm and *Schistosoma mansoni* in Mayuge district suggests that important gaps exist which are weakening the effectiveness of the MDA campaigns. One possible explanation for persistently high prevalence is re-infection among treated people. Several risk factors for hookworm [20–25] and schistosomiasis [26–28] infection/re-infection have been identified. These include gender, age, temperature, raising animals, WASH, exposure to contaminated environment, and lack of knowledge of parasitic infections. However, studies have presented conflicting results regarding risk factors for hookworm and schistosomiasis. There is also a lack of data in the context of MDA. This study sought to investigate whether hookworm and schistosomiasis are still prevalent in the context of > 15 years of an annual MDA program and additional NTD elimination programs, and to determine risk factors for prevalent infections.

## Methods

### Study area

This study was conducted in Mayuge district located in south-eastern Uganda, next to the shores of Lake Victoria and 146 km from Uganda’s capital, Kampala. Mayuge district has 14 sub-counties, including five lakeshore sub-counties: Bukabooli, Bukatube, Malongo, Wairasa, and Jaguzi. In a census conducted in 2020, as part of the MANE project, Mayuge’s population was 509,118, with 95,418 children under five and 156,182 children between five and 14 years old. As of January 2022, there were 504 primary schools, 41 health centres and one hospital.

### Study design and population

This was a cross-sectional study involving SAC from randomly selected primary schools in Mayuge district. A two-stage random sampling technique was used. In the first stage, the 504 schools in Mayuge district were listed by sub-county, and 33 were selected using probability proportional to size (PPS) [29], so that each school in a larger sub-county had a higher probability of selection, and each school in a smaller sub-county a lower probability of selection.

In the second stage, within each selected school, a class (40–50 children) was selected among the primary four and five classes (targeting nine to ten-year-old schoolchildren) using a table of random numbers. If only one class of this age group was present, that class was selected by default. All children in the selected classes were eligible for inclusion. Information about the study was provided to children and their parents/guardians in the local language, with the information for children adapted to ensure understanding. Children who signed the child assent form and whose parents/guardian provided written informed consent were included.

### Sample size

Sample size determination for the survey was based on the comparison of the prevalence of STH and schistosomiasis in Mayuge district in 2019 with the anticipated prevalence of STH and schistosomiasis after three years of project implementation. The baseline prevalence of STHs in 2019 was 15.8%, and it was anticipated that MANE program activities would reduce the prevalence of STHs to 12% by the time of the 2022 survey. For schistosomiasis, the prevalence was 27.2% in 2019, and it was estimated that MANE program activities would reduce schistosomiasis prevalence to 18% by the time of the 2022 survey.

Assuming the above changes in prevalence, a target sample size for the 2022 prevalence survey of at least 1,300 participants would be required for 80% power to detect a difference between 2019 and 2022 STH prevalence at 5% significance level. Assuming that each class contained at least 40 children, the number of selected schools (*n* = 33) was chosen in order to achieve the required sample size..

### Data collection and laboratory procedures

Field workers performing stool sample collection and survey questionnaires (supplementary file 1) were comprehensively trained before data collection, with training for standardised approaches to obtaining informed consent and assent, interviewing techniques, recording of responses, probing, and data handling/management. The research questionnaires were pre-tested and amended accordingly if necessary. Laboratory staff working at the public health centres in Mayuge district participated as field workers. Two field workers visited each school and conducted survey questionnaires with enrolled participants, entering data on assigned mobile phones.

The lead field worker cross-checked the completed questionnaires and wrote notes at the end of each day to ensure that all relevant questions had been asked and the responses adequately recorded. Field workers used mobile phones to record the data through the KoBoToolbox system and automatically saved and stored the data in the system when completing the questionnaire. The designated MANE project staff ensured appropriate supervision of the entire data collection process.

Each child was given a pre-labelled sterile stool container to collect a single stool sample and these were collected on the same day as the questionnaires were completed. These containers were then securely stored in cool boxes, which field workers transported to the district central laboratory at Kigandalo Health Centre IV, where the Kato-Katz examination method was employed [30]. Within 24 h of arrival at the laboratory, two slides were prepared from each stool sample. In cases where stool samples were not examined with the Kato-Katz method on the same day they were received, they were kept refrigerated at 4 °C overnight for examination the next day.

Examiners from the Vector-borne and Neglected Tropical Diseases Control Division (VCD) within the Ministry of Health of Uganda examined the prepared slides. Furthermore, a supervisor from the VCD double-checked 5% of the slides to confirm the accuracy and reliability of the results obtained from the initial examination.

### Potential risk factors

The following data on sociodemographic, lifestyle and environmental characteristics collected with the questionnaire were assessed as potential risk factors of interest: age, sex, location of school, household assets, type of latrine, open defecation (i.e. defecation in open fields/bush land etc.), water source for drinking, water source for other purposes (i.e. other than for drinking, such as bathing and washing clothes), water fetching time (time taken for round trip to water source from household: ≤ 30 min or > 30 min), household water facilities, hand-washing behaviour, soil contact, history of anthelminthic treatment, distance to and frequency of contact with freshwater, and knowledge of how hookworm and schistosomiasis can be acquired.

Participants’ school location was categorised into two groups: those who attended a school in a lakeshore sub-county and those who attended school in a non-lakeshore sub-county.

For water source variables, piped water connected to the dwelling or within the compound, piped water to neighbours, piped water to public places, boreholes, tube wells, protected dug wells, and protected springs were considered improved water sources. Unprotected dug wells, unprotected springs, lake water, river water, rainwater and unidentified water sources were considered unimproved. We categorised rainwater as a not-improved water source because rainwater in Mayuge district is usually harvested through gutters attached to roofs and house walls and exposed to contaminated soil.

For household assets, study participants were asked about types of home ownership and the possession of various household amenities, including cars, motorbikes, mobile phones, televisions, radios, access to electricity, and the availability of latrine facilities. We categorised participants into three groups based on their household assets: the "low" category was assigned to those with up to three household assets, the "moderate" category included those with four to five household assets, and the "high" category encompassed individuals with six to eight household assets.

### Statistical analysis

Data saved in the KoBoToolbox system were exported into Microsoft Office Excel, and checked for errors, missing values, and extreme values. The cleaned data were exported to Stata SE version 18.0 (Stata Corp; College Station, TX, USA) for statistical analysis.

The prevalence of STHs and schistosomiasis with a 95% confidence interval (CI) was calculated. The arithmetic egg count was obtained through the microscopic examination of slides prepared by applying the Kato-Katz method. A multiplication factor of 24 was applied to calculate eggs per gram since the stool quantity in a slide prepared with the Kato-Katz method contains 41.7 mg of stool (24 × 41.7 mg ≈ 1 g). The intensity of infection was categorised according to WHO guidelines [31].

The chi-square (χ2) test or Fisher’s exact test was applied to assess associations between categorical variables and the presence versus absence of hookworm infection and schistosomiasis. χ2 test for trend was used for evaluating associations with ordered categorical variables. Univariable logistic regression analysis was conducted to estimate crude odds ratios (OR) and 95% CI for the association between potential risk factors and hookworm infection and schistosomiasis. Multivariable logistic regression analysis was used to determine the association of risk factors with the prevalence of hookworm and schistosomiasis after controlling for confounders. In addition to age and sex, variables were selected for inclusion in the multivariable analysis based on the following criteria: 1) *p*-value for association with the outcome less than 0.1 in the univariable analysis; 2) biological plausibility for an association between an independent variable and the outcome.; 3) representative variable considering collinearity with similar variables.

The following variables were included in the multivariable analysis of risk factors for hookworm infection: sex, age, open defecation, water source used for non-drinking purposes, water fetching time, working in the soil, frequency of water-associated activities, and knowledge of skin penetration as a cause of parasite infection. The variable “water source used for drinking purposes” was not included in the multivariable analysis due to the possibility of collinearity with water sources used for non-drinking purposes.

For multivariable analysis of schistosomiasis, the following variables were included: sex, age, living near the lake or river, handwashing before eating, utilising improved water sources for other purposes, and the frequency of swimming, bathing, and washing clothes in risky environments. The variable “always handwashing after defecation” was excluded from the multivariable analysis due to collinearity with the variable of handwashing before eating. After completing the multivariable analysis, variables that exhibited a *p*-value ≤ 0.05 were regarded as statistically significant.

## Results

### Participant characteristics

**Fig. 1:**
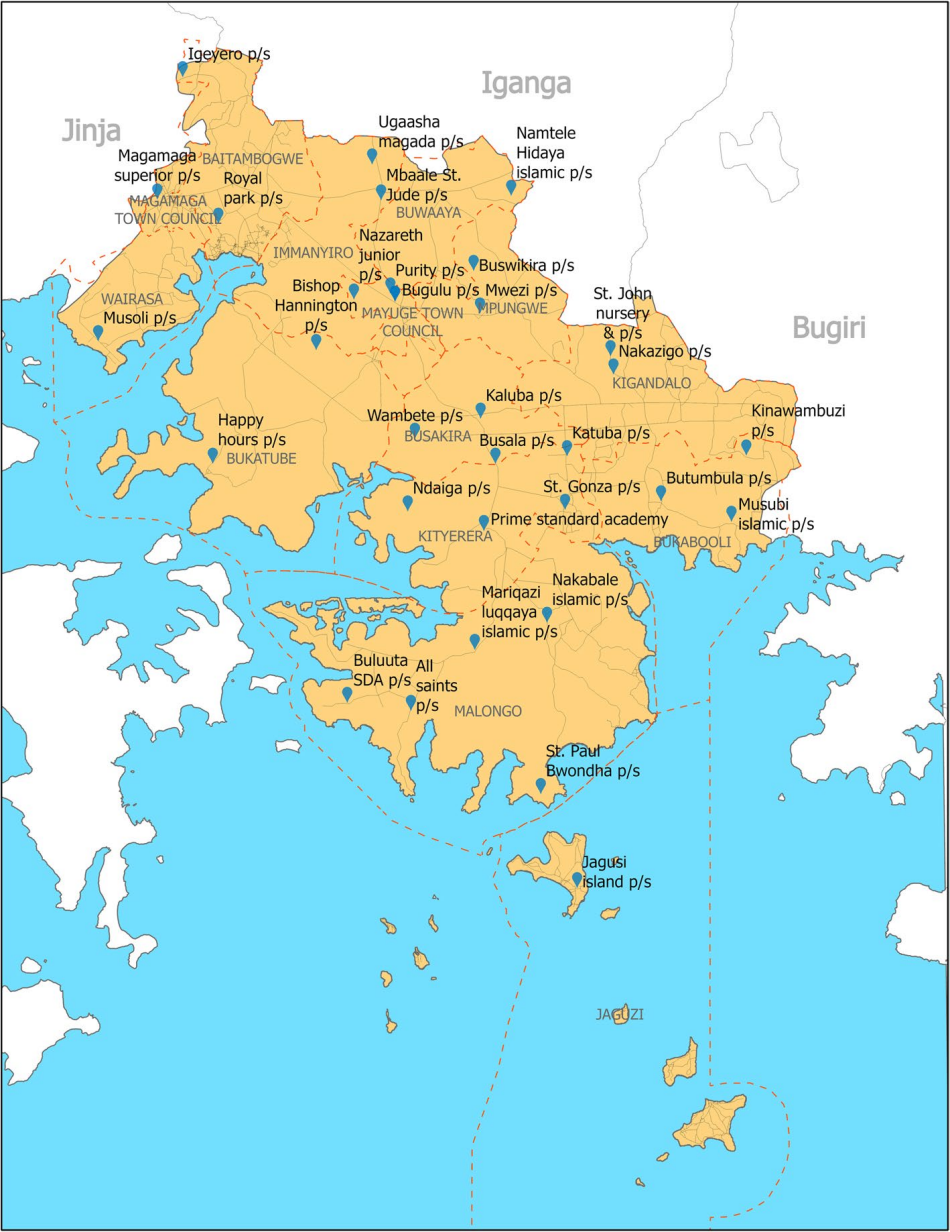
Location of the 33 selected schools for the study in Mayuge district

A total of 1,617 students from 33 schools (Fig. 1) were enrolled into the study, 632 (39%) from schools in lakeshore sub-countries. The mean age was 12.1 years (range 7.1–21.4 years), and 810 (50.1%) were male. Some respondents used more than one water source for drinking and other purposes; 9.2% (149) of all respondents used unimproved water sources for drinking, while 14.5% (234) used unimproved water sources for non-drinking purposes (Table 1). Borehole water was the primary source for drinking and other purposes in both lakeshore sub-counties and non-lakeshore sub-counties (supplementary Table 1). Of the study participants, 69.9% (1130) reported having hand-washing facilities at home. Among this group, 54.5% (616) had access to a fixed water facility within their homes.

**Table 1:** Prevalence, demographics, and intensity of hookworm infection and schistosomiasis

	Hookworm infection	Schistosomiasis
Infected population, N (%)	352 (21.8)	302 (18.7)
Infected population mean age, years (95% CI)	12.2 (12.1–12.4)	12.0 (11.8–12.2)
Infection intensity, N (%)		
Mild	338 (95.9)	177 (58.6)
Moderate	11 (3.1)	69 (22.8)
Heavy	3 (0.9)	56 (18.5)

### Prevalence results

Of 1,617 students, 361 (22.4%) were diagnosed with soil-transmitted helminth (STH) infection: 352 (21.8%) had hookworm infection, three (0.2%) had *Ascaris lumbricoides* infection, and six (0.4%) had *Trichuris trichiura* infection. The prevalence of hookworm infection by sub-county ranged from 8 to 39% (supplementary Table 2). Additionally, 302 students were found to be infected with *S. mansoni*, representing 18.7% of the students in the study (Table 1). The prevalence of schistosomiasis by sub-county ranged from 2 to 68% (supplementary Table 2). The prevalence of hookworm and *S. mansoni* co-infection was 5.8% (*n* = 94).

Infection intensity was categorized according to WHO definitions of light, moderate or heavy. Among the hookworm-positive cases, 338 children (95.9%) had mild-intensity, 11 (3.1%) had moderate-intensity, and three (0.9%) had heavy-intensity infection. On the other hand, among the *S. mansoni*-positive cases, 177 (58.6%) had mild-intensity, 69 (22.8%) had moderate-intensity, and 56 (18.5%) had heavy-intensity infection.

### Demographic characteristics, geological factors, WASH factors, and knowledge associated with hookworm infection and schistosomiasis

#### Crude associations with hookworm infection

There was no difference in hookworm prevalence between boys (184/810) and girls (168/807, OR: 0.89, 95% CI: 0.71–1.13, *p* = 0.35), and prevalence was similar across the different age groups. Hookworm infection was crudely associated with several WASH factors. Those using unimproved water sources, either for drinking purposes (OR: 1.58, 95% CI: 1.10–2.30, *p* = 0.02) or non-drinking purposes (OR: 1.43, 95% CI: 1.04–1.96, *p* = 0.03), had elevated hookworm prevalence. Other associated factors included extended water fetching time (> 30 min versus ≤ 30 min) (OR: 1.51, 95% CI: 1.16–1.97, *p* = 0.002), increased frequency of partaking in water-related activities, such as swimming, bathing, and washing clothes in lakes, rivers, and swamps (Trend OR: 1.27, 95% CI: 1.11–1.48, *p* = 0.001), and daily exposure to contaminated soil (working in the soil every day) (OR: 1.35, 95% CI: 1.03–1.77, *p* = 0.03). No significant associations were observed between hookworm infection status and other factors, including school location (lakeshore-locating vs. non-lakeshore-locating), household possession of appropriate latrines, handwashing practices, household assets, animal contact, deworming history, or knowledge regarding parasite infection.

#### Crude associations with schistosomiasis

The prevalence of schistosomiasis was slightly higher in boys (165/810) than in girls (137/807, OR: 0.80, 95% CI: 0.62–1.03, *p* = 0.08), and decreased somewhat with increasing age category (trend OR: 0.93, 95% CI: 0.86–1.00; *p* = 0.05).

Schistosomiasis was crudely associated with several environmental and behavioural factors. Students attending schools located along lakeshores had substantially higher odds of schistosomiasis (OR: 6.80, 95% CI: 5.01–9.24, *p* = 0.001). Additionally, utilising unimproved water sources for non-drinking purposes (OR: 1.62, 95% CI: 1.17–2.25, *p* = 0.01), not always washing hands after defecation (OR: 1.38, 95% CI: 1.08–1.78, *p* = 0.01), and before eating (OR: 1.74, 95% CI: 1.33–2.27, *p* = 0.001), were associated with having schistosomiasis. Frequency of partaking in water-related activities, such as swimming, bathing, and washing clothes in lakes, rivers, and swamps, was associated with schistosomiasis (Trend OR: 1.50, 95% CI: 1.29–1.75, *p* = 0.001) as was residing within one hour of water sources (OR: 1.92, 95% CI: 1.46–2.53, *p* = 0.001). However, no significant associations were observed between schistosomiasis and the use of appropriate latrines, open defecation behaviours, household assets, exposure to soil, or knowledge regarding parasite infections (Table 2).

**Table 2:** Univariable Analysis of Sociodemographic, Behavioural and Environmental Factors Associated with Hookworm and *Schistosoma mansoni* Infection

Factors		Total number (%)			Hookworm positive case; n(%)		Hookworm [OR(95%CI);*p*-value]				Schistosomiasis positive case; n(%)				Schistosomiasis [OR(95%CI);*p*-value]				
**Sex**																			
Male		810(50.1)			184 (22.7)		Reference				165(20.4%)				Reference				
Female		807(49.9)			168 (20.9)		0.89(0.71–1.13); *p* = 0.35				137(16.9%)				0.80(0.62–1.03); *p* = 0.08				
**Age (years)-category**																			
7–10		402(24.9)			78 (19.4)		Reference; *p* = 0.49				82(20.4)				Reference; *p* = 0.05				
11		307(19.0)			67 (21.8)		1.16(0.8–1.67)				72(23.5)				1.19(0.83–1.71)				
12		379(23.4)			86 (22.7)		1.22(0.86–1.72)				67(17.7)				0.83(0.59–1.2)				
13		329(20.4)			81 (24.6)		1.36(0.95–1.93)				50(15.2)				0.69(0.47–1.03)				
≥ 14		200(12.4)			40(20.0)		1.04(0.68–1.59)				31(15.5)				0.72(0.45–1.13)				
**Age (years)-general**							1.04(0.97–1.12);*p*= 0.25								0.93(0.86–1.00);*p*= 0.05				
**Household assets category**																			
Low(0–3)		595(36.8)			143(24.0)		Reference; *p* = 0.20				114(19.2)				Reference; *p* = 0.32				
Mid(4–5)		735(45.5)			154(21.0)		0.83(0.65–1.09)				127(17.3)				0.88(0.67–1.17)				
High(6–8)		287(17.8)			55(19.2)		0.75(0.53–1.06)				61(21.3)				1.14(0.8–1.61)				
**Hygiene factors**																			
Latrine type																			
Appropriate		778(48.1)			170(21.8)		Reference				156(20.1)				Reference				
None or Non appropriate		839(51.9)			182(21.7)		0.99(0.78–1.26); *p* = 0.94				148(17.6)				0.87(0.68–1.11); *p* = 0.27				
Open defecation(OD)																			
No		1057(65.4)			217(20.5)		Reference				196(18.5)				Reference				
Yes		560(34.6)			135(24.1)		1.23(0.96–1.57); *p* = 0.10				106(18.9)				1.02(0.79–1.33); *p* = 0.85				
I always wash my hands after defecation																			
Always		982(60.7)			205(20.9)		Reference				164(16.7)				Reference				
Not always		635(39.3)			147(23.2)		1.14(0.90–1.45); *p* = 0.28				138(21.7)				1.38(1.08–1.78); *p* = 0.01				
I always wash my hands before eating																			
Always		1190(73.6)			253(21.3)		Reference				194(16.3)				Reference				
Not always		427(26.4)			99(23.2)		1.12(0.86–1.46); *p* = 0.41				108(25.3)				1.74(1.33–2.27); *p* = 0.001				
**Water associated factors**																			
Lakeshore sub-country																			
No		985(60.9)			204(20.7)		Reference				75(7.6)				Reference				
Yes		632(39.1)			148(23.4)		1.17(0.92-1.49); *p*=0.19				227(35.9)				6.80(5.01-9.24); *p*=0.001				
Improved water source for drinking																			
Yes		1468(90.8)			308(20.9)		Reference				273(18.6)				Reference				
No		149(9.2)			44(29.5)		1.58(1.10-2.30); *p*=0.02				29(19.5)				1.05(0.69-1.62); *p*=0.79				
Improved water source for other purposes																			
Yes		1383(85.5)			288(20.8)		Reference				242(17.5)				Reference				
No		234(14.5)			65(27.4)		1.43(1.04-1.96); *p*=0.03				60(25.6)				1.62(1.17-2.25); *p*=0.01				
Water sources such as lake, river, swamp within one hour's walking distance from the house																			
No		649(40.1)			127(19.6)		Reference				86(13.3)				Reference				
Yes		968(59.9)			225(23.2)		1.24(0.97-1.59); *p*=0.08				218(22.5)				1.92(1.46-2.53); *p*=0.001				
Water-associated activities such as swimming, bathing, washing in lake, river, swamp																			
None		1168(72.2)			229(19.6)		Reference; *p*=0.01				182(15.6)				Reference; *p*=0.001				
Once a week		296(18.3)			77(26)		1.44(1.1-1.94)				69(23.3)				1.65 (1.2-2.25)				
Twice a week		61(3.8)			19(31.1)		1.86(1.06-3.25)				26(42.6)				4.02 (2.37-6.85)				
More than three a week		92(5.7)			27(29.3)		1.7(1.1-2.73)				25(27.1)				2.02 (1.24-3.29)				
**Soil associated factors**																			
I work in the soil every day																			
No		465(28.8)			85(18.3)		Reference				87(18.7)				Reference				
Yes		1152(71.2)			267(23.2)		1.35(1.03-1.77); *p*=0.03				215(18.7)				1.00(0.76-1.31); *p*=0.98				
I wear shoes when I go outside																			
Yes, always		771(47.7)			170(22.1)		Reference; *p*=0.17				128(16.6)				Reference; *p*=0.12				
Sometimes		391(24.2)			73(18.7)		0.89(0.68-1.18)				81(20.7)				1.29 (0.96-1.74)				
No		453(28.1)			109(23.9)		1.23(0.91-1.67)				93(20.4)				1.31(0.96-1.79)				
Water fetching time																			
≤ 30 min		1230(76.1)			246(20)		Reference				238(19.4)				Reference				
>30 min		387(23.9)			106(27.4)		1.51(1.16-1.97); *p*=0.002				64(16.5)				0.82(0.61-1.12); *p*=0.22				
**Knowledge factors**																			
I know swimming in lakes, rivers, swamp can cause parasite infection																			
Yes		1260(77.9)			263(20.9)		Reference				231(18.3)				Reference				
No		357(22.1)			89(24.9)		1.25(0.96-1.66); *p*=0.10				71(19.9)				1.1(0.82-1.48); *p*=0.50				
I know parasite can penetrate our feet																			
Yes		1290(79.8)			268(20.8)		Reference				242(18.8)				Reference				
No		327(20.2)			84(25.7)		1.32(0.99-1.75); *p*=0.06				60(18.3)				0.97(0.71-1.33); *p*=0.86				
** Deworming history previous year**																			
Yes		990(61.2)			218(22.0)		Reference				178(17.9)				Reference				
No		627(38.8)			134(21.4)		0.96(0.75-1.23); *p*=0.76				124(19.8)				1.12(0.88-1.45); *p*=0.37				

#### Multivariable logistic regression analysis for hookworm infection

In the multivariable analysis for hookworm infection (Table 3), children who reported that the journey time for fetching water was longer than 30 min had higher odds of hookworm infection compared to the ≤ 30 min group (AOR: 1.49, 95% CI: 1.13–1.96, *p* = 0.01). Those who reported working in the soil every day had increased odds of hookworm infection compared to those who did not (AOR: 1.36, 95% CI: 1.03–1.81, *p* = 0.03). Increased frequency of doing a water-associated activity was associated with higher odds of hookworm infection (AOR: 1.37, 95% CI: 1.01–1.86 and 1.67, 95% CI: 0.93–2.99 for once a week and twice a week, respectively, versus none) (Table 3).

**Table 3:** Multivariable analysis for the association between selected variables and hookworm infection

Factors	Hookworm [AOR(95%CI)]	*p*-value
Sex		
Male	Reference	
Female	0.95(0.75–1.21)	0.70
Age, per age-group increase	1.05(0.98–1.13)	0.18
Open defecation		
Yes	Reference	
No	0.88(0.68–1.14)	0.34
Improved water source for non-drinking purposes		
Yes	Reference	
No	1.06(0.74–1.53)	0.75
Water fetching time		
≤ 30 min	Reference	
> 30 min	1.49(1.13–1.96)	0.01
I work in the soil every day		
No	Reference	
Yes	1.36(1.03–1.81)	0.03
Swim, bath, washing in lake, river, swamp		
None	Reference	0.09
Once a week	1.37(1.01–1.86)	
Twice a week	1.67(0.93–2.99)	
More than three times a week	1.43(0.84–2.43)	
I know parasites can penetrate our feet		
Yes	Reference	
No	1.11(0.82–1.50)	0.50

#### Multivariable logistic regression analysis for schistosomiasis

In the multivariable analysis for schistosomiasis (Table 4), as age increased, the odds of schistosomiasis decreased somewhat (AOR: 0.93, 95% CI: 0.86–1.00, *p* = 0.05). Increased frequency of engaging in swimming, bathing, and washing activities in lakes, rivers, or swamps was associated with higher odds of schistosomiasis compared to those who never engaged in such activities (e.g. AOR: 2.91, 95% CI: 1.66–5.13 for twice a week versus none). Additionally, those not always washing hands before eating had higher odds of having schistosomiasis than those who always washed hands before eating (AOR: 2.00, 95% CI: 1.50–2.67, *p* = 0.001). Finally, those who lived within one hour by foot from a river, lake, or swamp had higher odds of schistosomiasis (AOR: 1.84, 95% CI: 1.35–2.50, *p* = 0.001) (Table 4).

**Table 4:** Multivariable analysis for identifying the association between selected variables and schistosomiasis

Factors	Schistosomiasis [AOR(95%CI)]	*p*-value
Sex		
Male	Reference	
Female	0.86 (0.66–1.11)	0.24
Age	0.93 (0.86–1.00)	0.05
Improved water source for other purposes		
Yes	Reference	
No	1.08 (0.73–1.58)	0.71
I wash my hands before eating		
Always	Reference	
Not always	2.00 (1.50–2.67)	0.001
Lake, river, and swamp within one hour by foot		
No	Reference	
Yes	1.84 (1.35–2.50)	0.001
Swim, bath, washing in lake, river, swamp		
None	Reference	0.003
Once a week	1.35 (0.96–1.89)	
Twice a week	2.91 (1.66–5.13)	
More than three times a week	1.46 (0.84–2.55)	

## Discussion

This study focused on factors influencing hookworm and schistosomiasis infection among SAC in Mayuge district, Uganda. Despite high program coverage of MDA for STH and schistosomiasis during the MANE project period (2019–2021), hookworm and schistosomiasis prevalence in 2022 remained high at 21.8% and 18.7%, respectively, compared to 15.1% and 27.2% in 2019. The study's findings improve understanding of the behavioural factors, environmental elements and sociodemographic traits that contribute to the pervasive presence of hookworm and schistosomiasis infections. These results may inform the interventions required for mitigating hookworm and schistosomiasis infections. As expected, activities associated with soil exposure, such as prolonged water fetching and frequent soil work, increased the likelihood of hookworm infection [32, 33]. Frequent engagement in water-related activities and poor hand-washing behaviour heightened the risk of schistosomiasis [32], whereas living farther away from water sources decreased the risk of contracting schistosomiasis [34].

The WHO recommends targeting SACs aged 9 to 10 years when conducting surveys to assess STH prevalence and schistosomiasis at the community level [31]. We adopted this approach in our study, so our findings do not necessarily represent all-age community prevalence. It is notable that in this SAC age group, the proportion of moderate to heavy schistosomiasis was higher than that of moderate to severe hookworm infection. This suggests that breaking the morbidity pattern for schistosomiasis when implementing WASH intervention may be more challenging than for hookworm infection.

Longer water fetching time was associated with hookworm infection, as found in an Ethiopia study [32]. We also found that daily work with exposure to soil was associated with hookworm infection. These findings are consistent with known mechanisms of infection. Hookworm prevalence increases in warm and moist soil and infective hookworm larvae can penetrate human skin directly, particularly the hands and feet [35]. In Mayuge District, SAC often engage in water-fetching practices without wearing footwear, heightening their vulnerability to hookworm larvae exposure during these activities. Therefore, this exposure can increase the likelihood of contracting hookworm infections among this age group.

The prevalence of schistosomiasis was lower for those who lived over an hour's walk from a river or lake and were attending schools in non-lakeshore sub-counties. This is because individuals who are not in close proximity to water bodies are less likely to come into contact with contaminated surface water where schistosomiasis-causing parasites may be present.[36]. Activities in the lake, river, and swamps, such as bathing, washing, and swimming, were associated with an increased risk of schistosomiasis, emphasising the well-established link between contaminated water and schistosomiasis infection [37].

Good hand hygiene practices in our study, specifically during pivotal daily activities—such as after defecation and before eating—were associated with reduced schistosomiasis. However, establishing a definitive cause-and-effect relationship between handwashing and schistosomiasis remains unclear. While *schistosoma* eggs in human excreta can release miracidia into freshwater, this parasite stage does not directly infect humans [38]. Similarly, the reasoning behind the association of schistosomiasis with handwashing before eating is difficult to explain. Further investigation is warranted to ascertain whether these factors causally contribute to schistosomiasis or if other unmeasured confounding variables influenced the observed association between handwashing behaviours and schistosomiasis.

We found no evidence of an association between hookworm infection and age, likely because our study focused on children attending school. Other researchers have indicated a discernible pattern of increasing hookworm infection rates with age, reaching a plateau typically observed in individuals in their thirties or forties [39, 40], which is different from other STHs. Studies that have examined the factors contributing to increased hookworm infection intensity with advancing age suggest that hookworms employ strategies to evade or suppress the host's immune response as individuals age [41, 42]. We observed a slight decline in schistosomiasis prevalence with increasing age in contrast to other studies, which have reported an increasing trend in schistosomiasis prevalence up to adolescence [16] or early adulthood followed by a decline in older age [43, 44].

We found no evidence of association between sex and helminth infections in our study, in contrast to multiple studies conducted in Africa (Ethiopia [45], Tanzania [46], Mali, Burkina Faso, Ghana [47], Cote d’Ivoire [47]) and Asia (Sri Lanka [48], Laos PDR [49]) that have identified higher prevalence of hookworm in males than females, predominantly due to greater exposure to soil among males [50, 51]. Similarly, schistosomiasis prevalence in Africa [52] generally shows male predominance, but this can be attributed to the gender disparity with water exposure, which may be less relevant among school-aged children. In some contexts, prevalence was higher in men due to activities such as swimming, farming, and fishing [50–53]; in others, prevalence was higher in women primarily due to contact with contaminated water for household chores [53].

Compared to the MANE 2019 baseline survey estimate of 27%, the prevalence of schistosomiasis decreased to 19%. However, contrary to the working hypothesis, the prevalence of hookworm increased slightly, from 16 to 22%. There are multiple possible explanations for this. First, we prepared two slides per stool sample for examination, aiming to enhance the detection yield of parasite eggs. However, in the prevalence survey conducted in 2019, only one slide per stool sample was examined. Consequently, the relatively increased prevalence of hookworm in this study compared to 2019 might have been influenced by this discrepancy in the number of slides per stool sample and greater detection in the follow-up relative to the baseline survey. Additionally, during the COVID-19 pandemic, the two-year closure of schools nationwide in Uganda resulted in increased participation of school-aged children residing in rural areas in labour alongside their parents [54, 55]. Male students became more involved in agricultural activities, while female students engaged more in domestic chores. These shifts in their daily routines likely led to increased exposure to contaminated soil, potentially contributing to the rise in hookworm infections in the 2022 prevalence survey compared to the hookworm prevalence in 2019. Therefore, the increase in the number of slides prepared per sample for examination and the increased exposure of school-aged children to contaminated soil due to the COVID-19 pandemic might explain the rise in hookworm infection prevalence despite the ongoing efforts of the MANE project.

### Limitation

Our study may have been subject to selection bias, due to the fact that only children attending school were eligible for inclusion, and they may not be representative of non-school attending children. Although every attempt was made to collect information on anticipated confounders, we cannot rule out the possibility of residual confounding. The study's respondents participated by responding in a self-reporting format, which could entail reliance on memory or reflect social desirability bias. As mitigation, when survey interviewers were trained, they were instructed to conduct one-on-one interviews with the interviewees and not to be subject to peer pressure. Additionally, standardised questions were posed to the interviewees, and interviewers recorded responses directly into standardised templates using mobile phones. This approach aimed to mitigate the limitations associated with self-reporting.

## Conclusions

Longer water fetching times and daily work in the soil are identified as common risk factors for hookworm infection. The association between water fetching time and hookworm infection underscores the importance of efficient water supply systems to minimise exposure to contaminated environments. Daily soil work highlights the need for protective measures, such as appropriate footwear, to reduce the risk of hookworm infection.

Schistosomiasis is associated with exposure to contaminated water sources, emphasising the critical role of access to safe and clean water in preventing the disease. Inadequate handwashing behaviours are linked to an increased risk of schistosomiasis, which needs further investigation to identify a cause-and-effect relationship between handwashing and schistosomiasis.

The study underscores the significance of effective water supply systems and protective measures in reducing hookworm infection risks, as well as the critical role of safe water access in preventing schistosomiasis. Further investigation is needed to understand the relationship between handwashing practices and schistosomiasis risk. These findings emphasise the importance of Water, Sanitation, and Hygiene (WASH) interventions in addressing both hookworm and schistosomiasis. Integrated neglected tropical disease elimination programs should prioritise WASH improvements to reduce exposure to contaminated soil and water, highlighting the interconnected nature of environmental factors, hygiene behaviours, and disease transmission..

### Supplementary Information

Supplementary Material 1.

Supplementary Material 2.

Supplementary Material 3.

## Data Availability

Data is provided as a supplementary file.

## Abbreviations

AOR: Adjusted Odds Ratio
CI: Confidence Interval
CR: Cure rate
DALY: Disability-adjusted life years
HC: Health centre
KOICA: Korean International Cooperation Agency
MANE project: Mayuge NTDs Elimination project
MDA: Mass drug administration
NTDs: Neglected tropical diseases
OR: Odds Ratio
PPS: Probability proportional to size
PSAC: Preschool-aged children
PZQ: Praziquantel
STH: Soil-transmitted helminth
VCD: Vector-borne and NTD control division
WASH: Water, sanitation, and hygiene
WHO: World Health Organization
