# Supplementary material for: Factors associated with hookworm and Schistosoma mansoni infections among school-aged children in Mayuge district, Uganda
Source: BMC Public Health. 2024 Jun 18;24:1620. doi: 10.1186/s12889-024-19092-7 (PMC11184691; doi:10.1186/s12889-024-19092-7)
Supplement: Supplementary file 2 — Supplementary Material 2. [file 12889_2024_19092_MOESM2_ESM.pdf]

(Supplementary Table 1) Water sources for drinking purpose and other purpose in lakeshore sub-county and non-lakeshore sub-county of Mayuge district.

Purpose of water use	water source	Lakeshore sub-county	Non-lakeshore sub-county
For drinking; n (%)	Piped water(into dwelling)	4(0.6)	48(4.9)
	Piped water (neighbour)	4(0.6)	18(1.8)
	Piped water (share in the community)	64(10.1)	23(2.3)
	Borehole	368(58.2)	720(73.1)
	Protected dug well	63(10.0)	78(7.9)
	Protected spring	17(2.7)	61(6.2)
	Unprotected dug well	61(9.7)	23(2.3)
	Unprotected spring	12(1.9)	4(0.4)
	Lake or river water	38(6.0)	8(0.8)
	Rain water or other	1(0.2)	2(0.2)
Total		632(100)	985(100)
For other purpose; n (%)	Piped water(into dwelling)	5(0.8)	49(5.0)
	Piped water (neighbour)	4(0.6)	16(1.6)
	Piped water (share in the community)	41(6.5)	20(2.0)
	Borehole	340(53.8)	681(69.1)
	Protected dug well	54(8.5)	91(9.2)
	Protected spring	21(3.3)	61(6.2)
	Unprotected dug well	77(12.2)	47(4.8)
	Unprotected spring	17(2.7)	10(1.0)
	Lake or River water	71(11.2)	8(0.8)
	Rain water	2(0.3)	1(0.1)
	Other	0(0)	1(0.1)
Total		632(100)	985(100)
