# Supplementary material for: Factors associated with hookworm and Schistosoma mansoni infections among school-aged children in Mayuge district, Uganda
Source: BMC Public Health. 2024 Jun 18;24:1620. doi: 10.1186/s12889-024-19092-7 (PMC11184691; doi:10.1186/s12889-024-19092-7)
Supplement: Supplementary file 3 — Supplementary Material 3. [file 12889_2024_19092_MOESM3_ESM.pdf]

(Supplementary Table 2) Hookworm and schistosomiasis prevalence by sub-county of Mayuge district, Uganda

	Sub-county														Total
	1	2	3	4	5	6	7	8	9	10	11	12	13	14	
The number of selected schools (n)	3	2	1	6	1	2	3	1	2	3	4	1	2	2	33
Hookworm prevalence§ (%)	39	12	22	22	10	9	14	20	22	22	36	6	8	29	21.8
Schistosomiasis prevalence§ (%)	44	21	26	34	68	8	8	2	5	10	11	2	3	8	18.7

Lakeshore sub-county: 1 Bukabooli, 2 Bukatube, 3 Jagusi, 4 Malongo, 5 Wairasa,

Non-lakeshore sub-county: 6 Baitambogwe, 7 Busakira, 8 Buwaaya, 9 Imanyiro, 10 Kigandalo, 11 Kityerera, 12 Magamaga T/C, 13 Mayuge T/C, 14 Mpungwe

§ mean percentage of school-level population
